# Cemento-Ossifying Fibroid Epulis in the Posterior Maxilla

**DOI:** 10.7759/cureus.46167

**Published:** 2023-09-28

**Authors:** Vijaya Lakshmi G, A Saneem Ahamed, M.R.C Rajeswari, Prem Karthick, A Jayasenthil

**Affiliations:** 1 Department of Oral and Maxillofacial Surgery, Priyadarshini Dental College and Hospital, Chennai, IND; 2 Department of Oral Pathology and Microbiology, Priyadarshini Dental College and Hospital, Chennai, IND; 3 Department of Conservative Dentistry and Endodontics, Priyadarshini Dental College and Hospital, Chennai, IND

**Keywords:** periodontal ligament, calcifications, fibroma, ossifying, cemento-ossifying fibroma, gingiva

## Abstract

Cemento-ossifying fibroma is a benign fibro-osseous lesion arising from the periodontal ligament and has the potential to form cementum and bone in the periodontal ligament. Cemento-ossifying fibroma is a painless, pedunculated, or sessile, smooth exophytic growth arising attached to the gingival tissues. We present a case of cemento-ossifying fibroid epulis in the posterior maxilla attached to the interdental gingiva between the 26 and 27 region buccally in a 52-year-old female patient managed with surgical excision of the lesion, extraction of the involved teeth, curettage, and palatal obturator while under general anesthesia. The patient was followed up post-operatively, healing was satisfactory, there were no signs of infection, and no recurrence was noted in the six-month follow-up period.

## Introduction

Cemento-ossifying fibroid epulis is a benign fibro-osseous lesion. It is defined as a well-demarcated and occasionally encapsulated lesion comprising fibrous tissue containing variable amounts of mineralized material resembling bone (ossifying fibroma), cementum (cementifying fibroma), or both [1-3]. Subgingival plaque and calculus, ill-fitting dental appliances, dental biofilm, and irregular restorations provoke the lesion [4,5]. Peripheral ossifying fibroma is the third most common focal reactive overgrowths and accounts for about 27% of cases and is seen more commonly in the younger age group, predominantly in the second and third decades [6]. Peripheral ossifying fibroma is presumed to emerge from the periodontal ligament as it occurs on the gingiva and contains oxytalan fibers dispersed among the calcified layers [6]. The cells in the periodontal ligament are capable of forming cementum and bone [6]. Peripheral cemento-ossifying fibroma accounts for 3.1% of all oral tumors and 9.6% of gingival lesions [1]. The lesion has a high female predilection [1]. Fibrous epulis has a smooth surface, grows exophytically, exhibits the color of mucosa, and is asymptomatic and occasionally ulcerated [7]. The lesion had a nodular presentation in 98.4%, 60.4% of the cases were pedunculated, 84.7% were asymptomatic, and few lesions increased to a large size [5]. Cemento-ossifying fibroma appears as radio-opaque foci of calcification or doesn’t exhibit any radiographic findings which is attributed to the mineral content [8]. Cemento-ossifying fibroma is a reactive lesion occurring on the gingiva and is differentiated from the ossifying fibroma which is intraosseous and a true neoplasm [6]. Cemento-ossifying fibroid epulis is treated by surgical excision including periodontal ligament periosteum and scaling of adjacent teeth [1]. This paper highlights the clinical presentation and surgical management of cemento-ossifying fibroid epulis in the maxillary posterior region treated in our center.

## Case presentation

**Figure 1:**
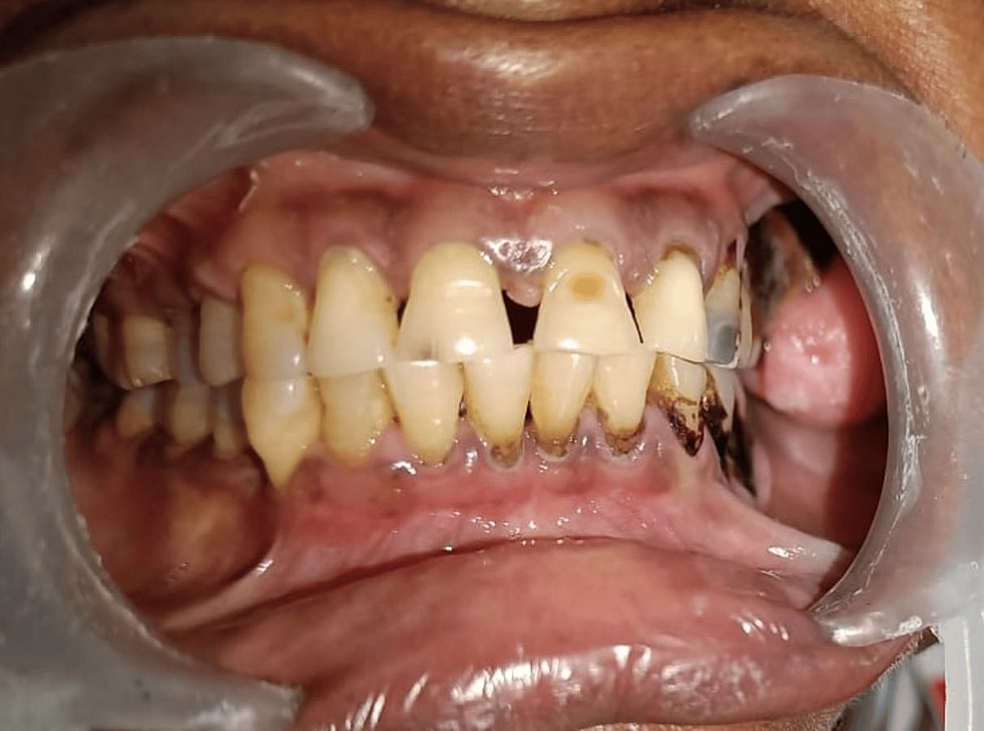
Pre-op clinical image 3x2 cm solitary swelling was noted in relation to 26, 27 region buccally, which is oval in shape, pedunculated attached to the interdental gingiva between 26 and 27 region and pink in color and has smooth texture.

A 52-year-old female patient reported to the Department of Oral and Maxillofacial Surgery with the chief complaint of swelling in the upper back teeth region for one month. The patient gave a history of swelling for three years which gradually progressed to the current size. No history of pain or discharge from the swelling. Past medical history of the patient reveals that the patient has a known case of diabetes and has been under medication for the same for 15 years. On clinical examination, 3x2 cm solitary swelling was noted in the 26, 27 region buccally, which is oval in shape, pedunculated attached to the interdental gingiva between the 26 and 27 region and pink in color and has smooth texture; no discharge noted (Figure 1).

**Figure 2:**
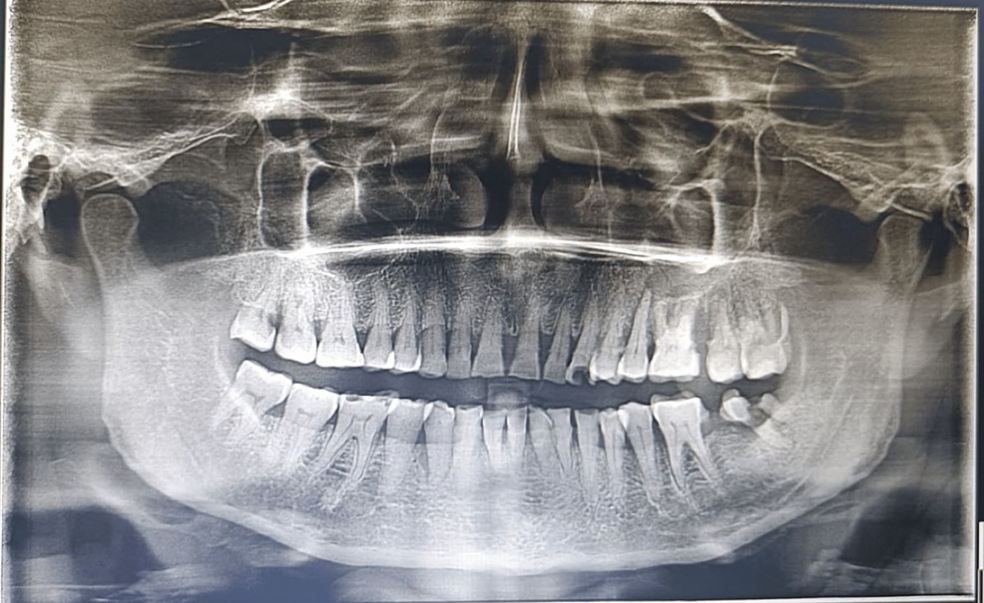
Pre-operative OPG OPG reveals a radiopaque rim surrounding the radiolucent region superimposed over the 26, 27, and 28 region. OPG: Orthopantomogram

There was no bony expansion noted clinically. All inspectory findings were confirmed by palpating; swelling was firm in consistency, mobile, non-tender, non-compressible, and pedunculated. The patient was provisionally diagnosed with peripheral ossifying fibroma. Orthopantomogram (OPG) and intraoral periapical radiography (IOPA) were taken which revealed a radiopaque rim surrounding the radiolucent region superimposed over 26, 27 and 28 region (Figure 2).

**Figure 3:**
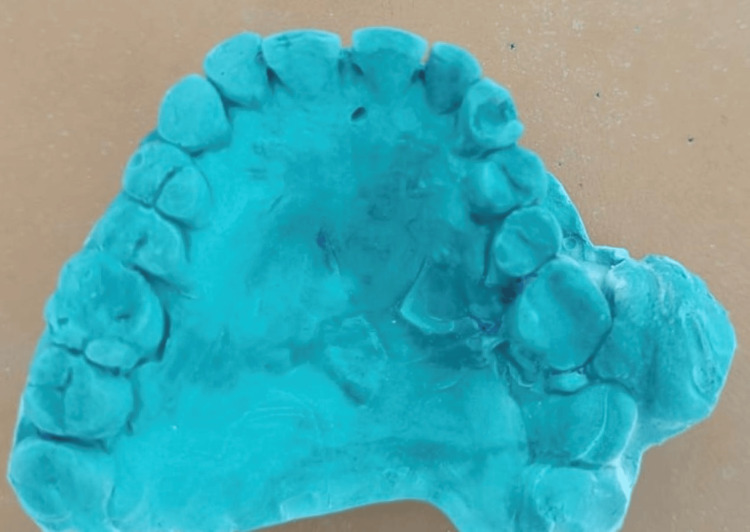
Model of the lesion The maxillary cast shows a pedunculated lesion attached to the interdental gingiva between the 26 and 27 region.

**Figure 4:**
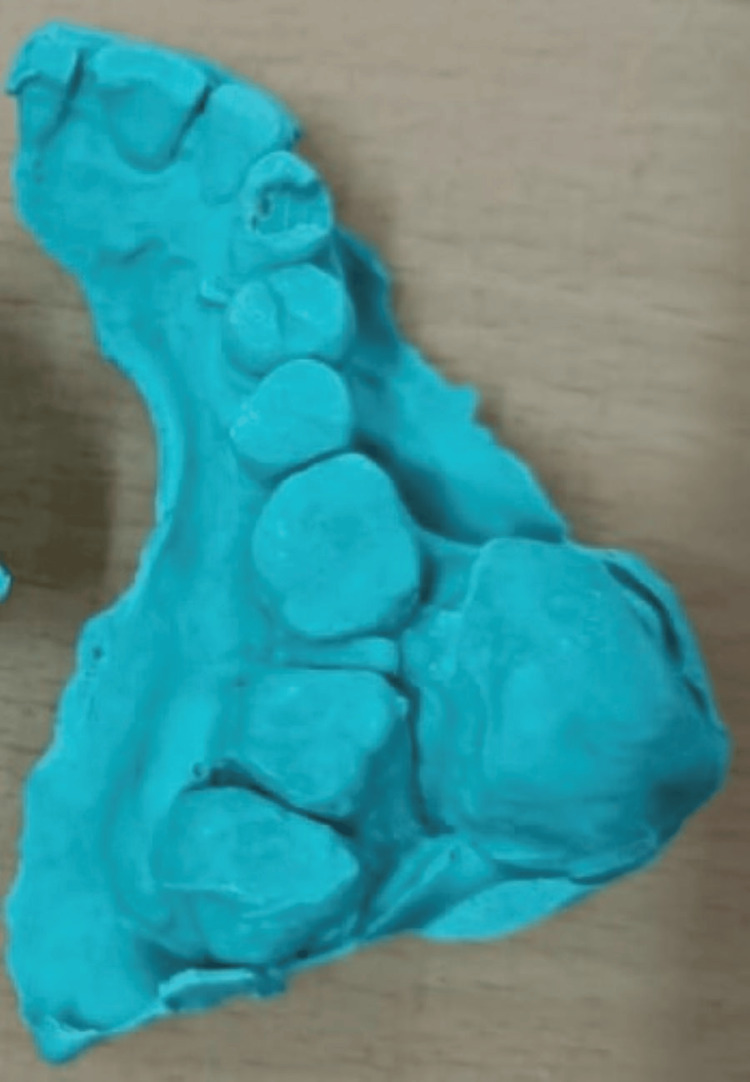
Model of the lesion The maxillary cast on a lateral aspect shows that a 3x2 cm solitary swelling was noted in the 26 and 27 region buccally.

The patient was planned for surgical excision of the lesion, extraction of 25, 26, 27, and 28 region, and palatial obturator. Written informed consent was obtained from the patient. Upper and lower impressions were made, and models were made (Figures 3, 4).

**Figure 5:**
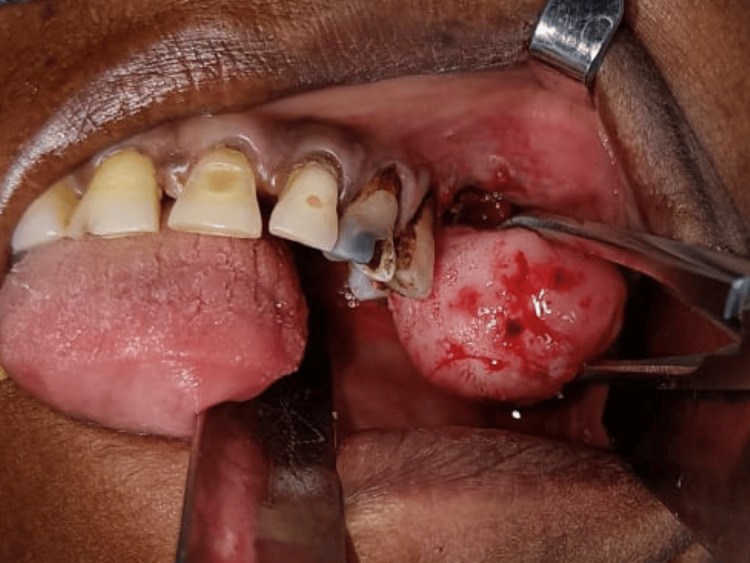
Excision of the lesion Surgical excision of the lesion was performed along with the pedicle and the interdental gingiva.

**Figure 6:**
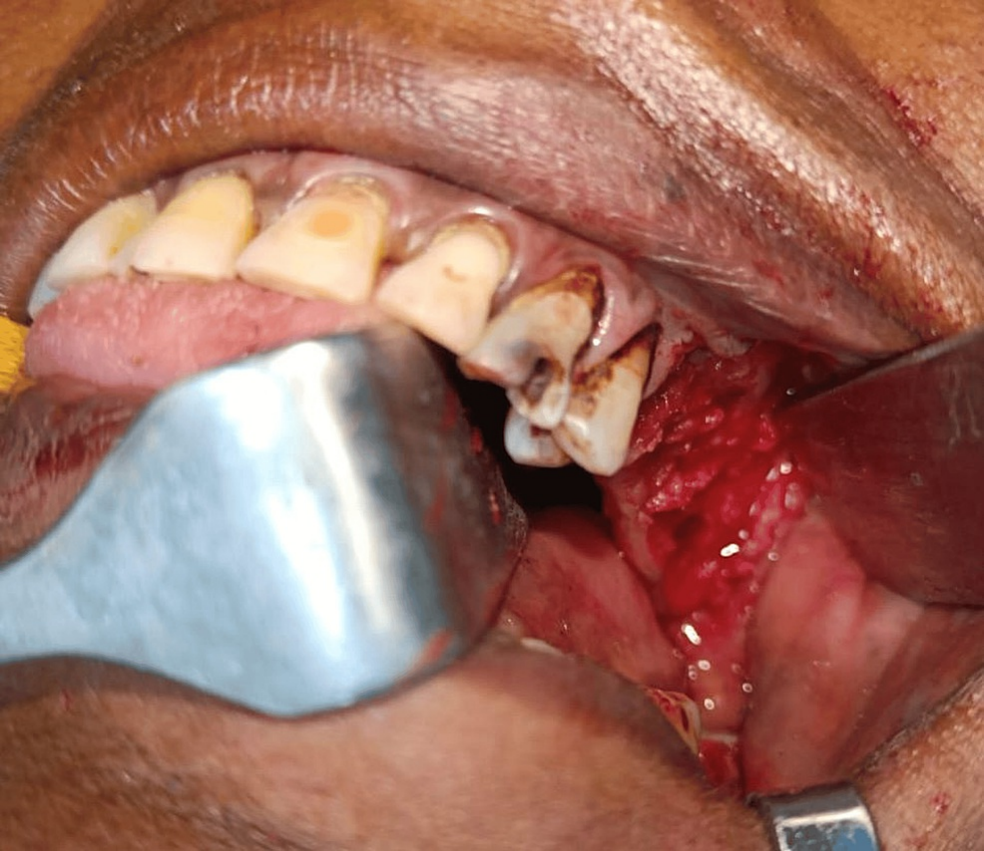
Osteoplasty post excision Osteoplasty was performed after excision and bone was filed.

**Figure 7:**
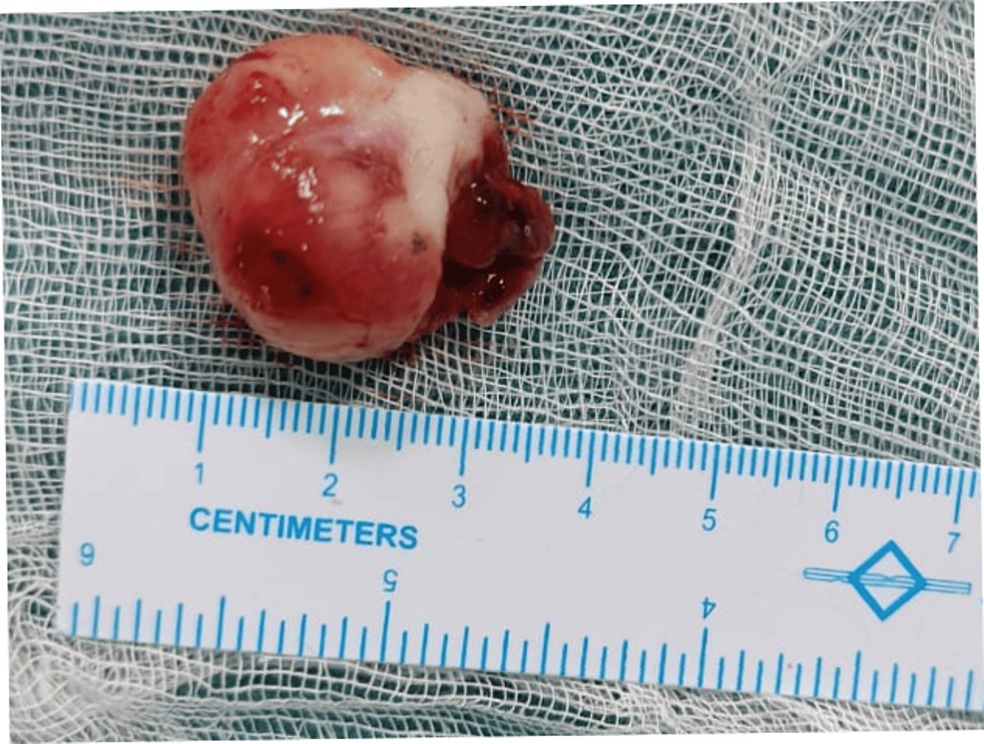
Excised specimen Excised lesion along with the pedicle.

A palatal obturator was fabricated extending over the alveolus to the buccal vestibule of the 25-28 region. The planned procedure was carried out under general anesthesia, extraction of 25, 26, 27, and 28 was performed, the lesion was surgically excised (Figure 5), complete curettage and osteoplasty were done to remove 2-3 mm of bone using bone burs, bone was smoothened using bone files, and hemostasis was achieved using direct pressure (Figure 6). Figure 7 shows the excised specimen.

**Figure 8:**
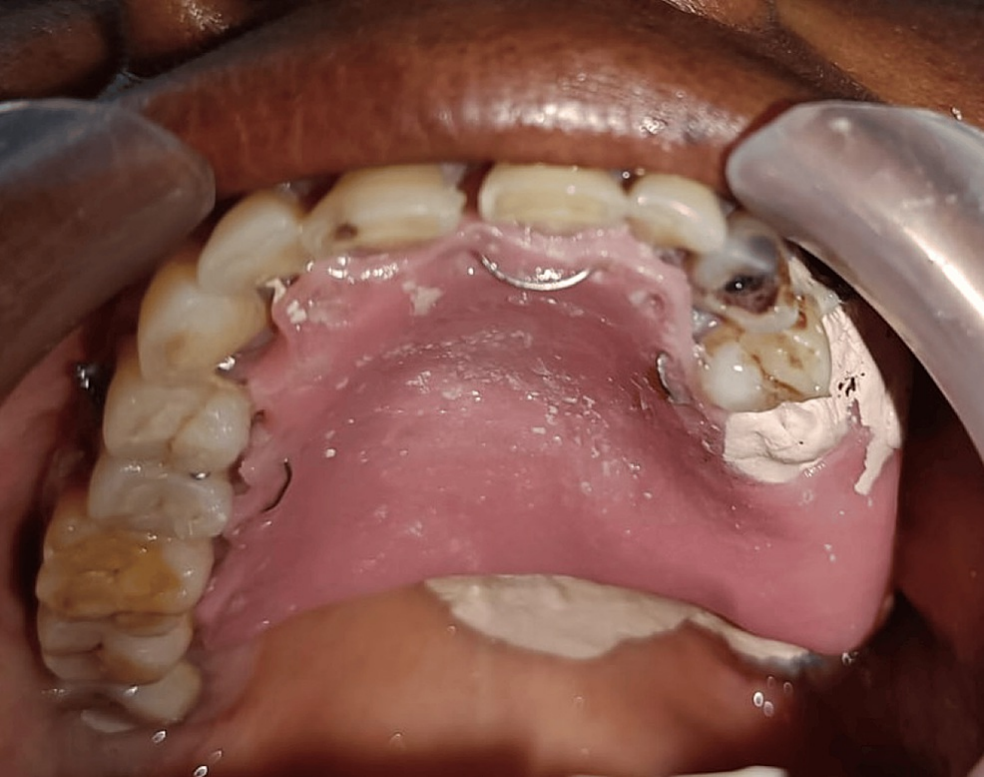
Palatal obturator in situ Coe-pack was placed in the operated site, the prepared palatal obturator was adapted and secured to the teeth using 26-gauge stainless steel wires.

Closure was done by raising a buccal advancement flap and simple interrupted sutures were placed using the 3-0 Vicryl suture material. Coe-pack was placed in the operated site, the prepared palatal obturator was adapted and secured to the teeth using 26-gauge stainless steel wires (Figure 8).

The specimen was sent for histopathological examination, suggestive of cemento-ossifying fibroid Epulis (fibroepithelial polyp with calcification). The obturator was removed after the second post-operative week. Healing was satisfactory.

## Discussion

Cemento-ossifying fibroid epulis is a benign fibro-osseous lesion. It is defined as a well-demarcated and occasionally encapsulated lesion comprising fibrous tissue containing variable amounts of mineralized material resembling bone (ossifying fibroma), cementum (cementifying fibroma), or both [1-3]. Shepherd [9] was the first to describe this lesion as alveolar exostosis which was later modified by Eversole and Robin as peripheral ossifying fibroma [10]. The lesion is also referred to as peripheral cemento-ossifying fibroma, ossifying fibro-epithelial polyp, peripheral fibroma with cementogenesis, peripheral fibroma with osteogenesis, calcifying or ossifying fibroma epulis, peripheral fibroma with calcification, and calcifying fibroblastic granuloma [5]. Peripheral ossifying fibroma is the third most common focal reactive overgrowths and accounts for about 27% of cases and is seen more commonly in the younger age group, predominantly in the second and third decades [6]. Only 0.5% of the cases were reported in the older age group [4]. Peripheral ossifying fibroma is presumed to emerge from the periodontal ligament as it occurs on the gingiva and contains oxytalan fibers dispersed among the calcified layers [6]. The cells in the periodontal ligament are capable of forming cementum and bone [6]. The patient does not seek treatment as the lesion is asymptomatic which leads to progression for long periods [1]. Our patient gave history of the presence of the lesion for three years since she reported. Two different schools of thought were proposed to understand the histogenesis of peripheral ossifying fibroma [4]. One is the pyogenic granuloma undergoes progressive fibrous maturation and calcification to form peripheral ossifying fibroma, and the other is pyogenic ossifying fibroma is the result of inflammatory hyperplasia of cells of periodontal ligament or periosteum. Metaplasia of connective tissue leads to dystrophic calcification and bone formation [4].

Some genetic conditions are associated with multiple peripheral ossifying fibromas like multiple endocrine neoplasia- type II, nevoid nasal cell carcinoma syndrome, neurofibromatosis, and Gardner syndrome [4]. Clinically, peripheral ossifying fibroma presents as smooth lobulated pink mass either pedunculated or sessile [4]. The lesion is asymptomatic initially and progresses to a size that causes pain, functional alteration, and cosmetic deformity [1]. Our patient had 3x2 cm solitary swelling in the 26, 27 region buccally, which is oval in shape, pedunculated attached to the interdental gingiva between the 26 and 27 region and pink in color and has smooth texture, and no discharge was noted. There was no bony expansion was noted clinically. The swelling was firm in consistency, mobile, non-tender, and non-compressible. Peripheral cements-ossifying fibroma shows similar clinical features to other extraosseous lesions [1]. It is frequently misdiagnosed as pyogenic granuloma, peripheral giant cell granuloma, fibrous dysplasia, osteoid osteoma, osteoblastoma, low-grade osteosarcoma, cementoblastoma, chronic osteomyelitis, and sclerosing osteomyelitis of Garre [1]. Cemento-ossifying fibroma appears as radio-opaque foci of calcification or does not exhibit any radiographic findings which is attributed to the mineral content [8]. OPG and IOPA were taken in our patient which revealed a radiopaque rim surrounding the radiolucent region superimposed over the 26, 27, and 28 region.

**Figure 9:**
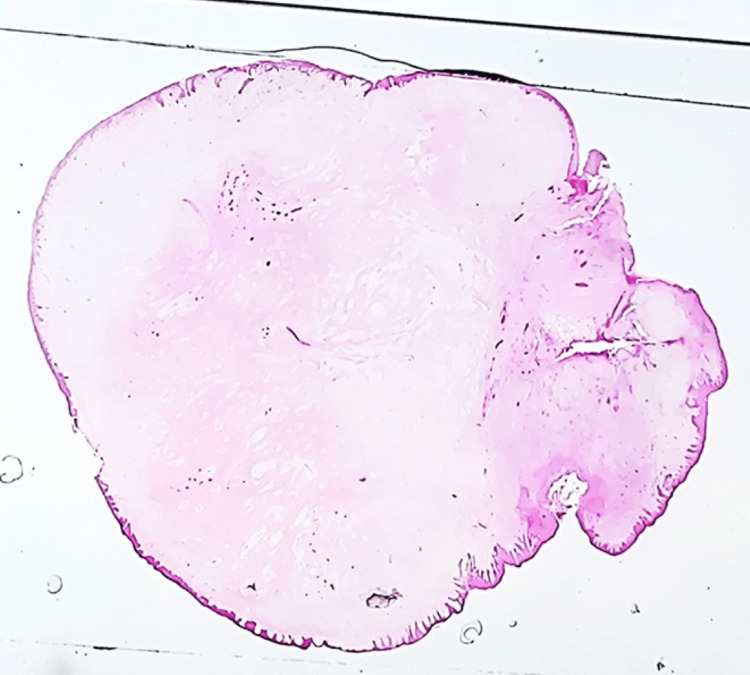
Histopathology is suggestive of cemento-ossifying fibroid epulis (fibroepithelial polyp with calcification) The fibrous connective tissue shows dense interlacing bundles with fibroblasts and vascular spaces. Bony trabecular and cementum-like mass seen with the center of the lesion.

**Figure 10:**
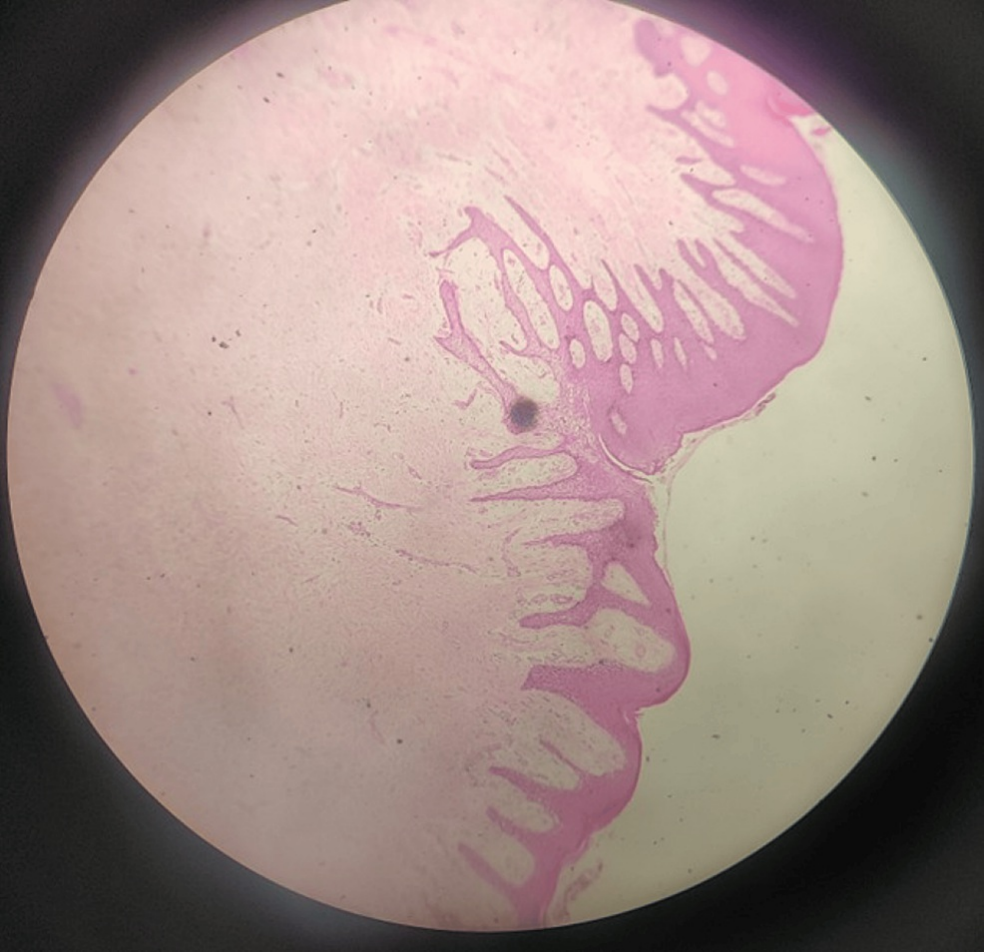
Histopathology is suggestive of cemento-ossifying fibroid epulis (fibroepithelial polyp with calcification) Parakeratinized stratified squamous epithelium with long and slender rete ridges. Fibrocellular connective tissue with calcifications.

Histopathological examination of the lesion reveals parakeratinized stratified squamous epithelium with long and slender rete ridges. There was fibrocellular connective tissue with calcifications. Cellular areas consist of fibroblasts with trabecular bone lined by osteoblasts. Multiple round-to-oval hematoxoyophilic calcified matrix is present resembling a cementum-like material [8]. Histopathology in our case represented dense interlacing bundles of fibrous connective tissue with covering epithelium. The fibrous connective tissue shows dense interlacing bundles with fibroblasts and vascular spaces. There are areas of bony trabecular and cementum-like mass seen at the center of the lesion. There is focal collection of chronic inflammatory cells like plasma cells and lymphocytes seen in some areas. The covering epithelium is hyperplasticity stratified squamous epithelium, showing thin long reteridges in most of the areas. Histopathology is suggestive of cemento-ossifying fibroid epulis (fibroepithelial polyp with calcification) (Figures 9, 10).

Peripheral ossifying fibroma needs to be differentiated from other reactive gingival lesions such as pyogenic granuloma, peripheral giant cell granuloma, and peripheral odontogenic fibroma. Pyogenic granuloma clinically appears as a red mass with an ulcerated surface and exhibits vascular proliferation resembling granulation tissue under a microscope. Peripheral giant cell granuloma shows giant cells scattered in fibrous stroma. Peripheral odontogenic fibroma contains prominent islands of odontogenic epithelium. Bony involvement is noted in few cases such as erosion of superficial bone, foci of calcifications, widening of periodontal ligament space, thickening of lamina dura, and teeth getting migrated with loss of interdental bone [4].

**Figure 11:**
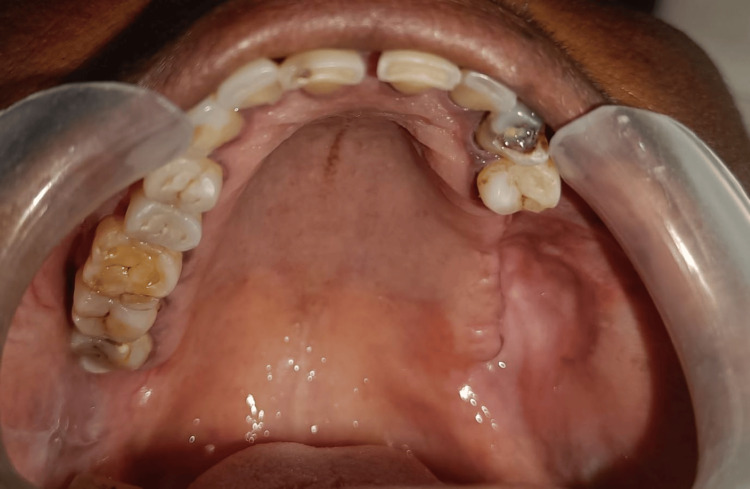
Post-operative image after excision Post-op image shows satisfactory wound healing.

**Figure 12:**
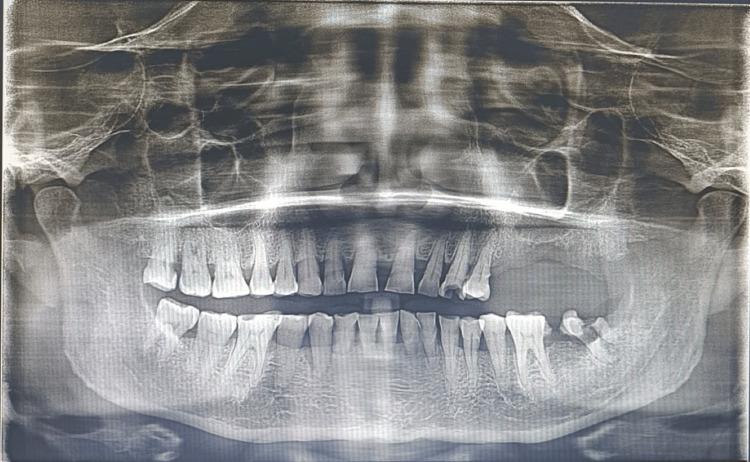
Post-operative OPG Post-operative OPG shows no recurrence in the operated site in the six-month follow-up.

Treatment comprised conservative surgical excision and scaling of adjacent teeth. The recurrence rate of peripheral cemento-ossifying fibroma is high and is probably due to incomplete removal of the lesion, repeated injury, or persistence of local irritants [8]. Excision can be carried out using electrosurgery or lasers. Hemostasis was better when excision was carried out using electrosurgery compared to a scalpel. Disadvantages were thermal injury and delayed healing. Laser surgical excision was good as their healing was better, good acceptance by the patients and there was not any compromise in the histological diagnosis [11]. In our case procedure was carried out under general anesthesia, extraction of 25, 26, 27, and 28 was performed, the lesion was surgically excised, complete curettage and osteoplasty were done and hemostasis was achieved. Closure was done by raising a buccal advancement flap and sutures were placed using 3-0 Vicryl. Coe-pack was placed in the operated site, the prepared palatal obturator was adapted and secured to the teeth using 26-gauge stainless steel wires. The obturator was removed after the second post-operative week. Healing was satisfactory. The patient was assessed clinically (Figure 11) and radiographically (Figure 12) for the presence of recurrence at six months post-operatively, and no recurrence was noted.

Recurrence was reported by many authors in the literature such as Cundiff observed 16%, Eversole and Robin reported 20%, and 8.9%-20% by Bhaskar and Jacoway [10,12,13]. Recurrence might be attributed to incomplete removal of the lesion and local irritation such as plaque and calculus [8].

## Conclusions

Cemento-ossifying fibroid epulis is common in females. Local irritants like plaque and calculus are the main causative factors. The lesion is exophytic, firm in consistency, smooth textured, and asymptomatic in nature which delays the patient seeking treatment. Such a lesion must be identified and diagnosed during routine clinical examination of the oral cavity. Prompt diagnosis and surgical excision of the lesion with the pedicle and interdental gingiva and osteoplasty with removal of involved teeth might prevent recurrence of the lesion. Surgical excision with a scalpel gives good margins for histopathological examination but causes bleeding in the operating site, which may be avoided by using lasers. Osteoplasty might aid in reducing the recurrence rates. Long-term follow-up is needed to rule out any recurrence.
